# Effect of Stepped Care on Sexual Interest and Enjoyment in Distressed Patients with Head and Neck Cancer: A Randomized Controlled Trial

**DOI:** 10.1016/j.esxm.2020.100304

**Published:** 2021-01-15

**Authors:** Laura E.R. Schutte, Heleen C. Melissant, Femke Jansen, Birgit I. Lissenberg-Witte, C. René Leemans, Mirjam A.G. Sprangers, Marije R. Vergeer, Irma M. Verdonck-de Leeuw, Ellen T.M. Laan

**Affiliations:** 1 Department of Sexology and Psychosomatic Gynaecology, Amsterdam University Medical Centers, Location AMC, University of Amsterdam, Amsterdam, The Netherlands; 2 Department of Clinical, Neuro- and Developmental Psychology, Faculty of Behavioral and Movement Sciences, Amsterdam Public Health Research Institute, Vrije Universiteit Amsterdam, Amsterdam, The Netherlands; 3 Department of Otolaryngology-Head and Neck Surgery, Cancer Center Amsterdam, Amsterdam University Medical Centers, Location VUmc, Vrije Universiteit Amsterdam, Amsterdam, The Netherlands; 4 Department of Epidemiology and Biostatistics, Amsterdam University Medical Centers, Location VUmc, Vrije Universiteit Amsterdam, Amsterdam, The Netherlands; 5 Department of Medical Psychology, Amsterdam University Medical Centers, Location AMC, University of Amsterdam, Amsterdam, The Netherlands; 6 Department of Radiation Oncology, Amsterdam University Medical Centers, Location VUmc, Vrije Universiteit Amsterdam, Amsterdam, The Netherlands

**Keywords:** Head and neck cancer, RCT, Stepped care, Quality of life, Distress, Sexuality

## Abstract

**Introduction:**

A recent randomized controlled trial (RCT) in patients with head and neck cancer (HNC) with psychological distress showed that a stepped care (SC) program targeting psychological distress compared with care as usual (CAU), is (cost)effective in reducing psychological distress.

**Aim:**

The aim of the present study was to investigate whether SC can coalleviate problems with sexual interest and enjoyment. A secondary aim was to investigate whether the presence of an unmet sexual health need and having a psychiatric disorder (depression or anxiety) at baseline moderated any effect of SC on these sexual variables until 1-year follow-up.

**Methods:**

HNC survivors (*N* = 134), randomized to SC or CAU, were assessed regarding their sexual interest and enjoyment before and after the intervention and at 3, 6, 9, and 12 months follow-up. Linear mixed models were used to evaluate differences in the course of sexual interest and enjoyment between SC and CAU.

**Main Outcome Measure:**

The “sexuality” symptom subscale, part of the European Organization for Research and Treatment of Cancer, Quality of Life Questionnaire, Head and Neck Cancer–specific module.

**Results:**

Of all patients, 76.1% had an unmet sexual need at baseline, 24.6% had a psychiatric disorder (anxiety or depression). SC did not reduce problems with sexual interest and enjoyment at any of the follow-up measurements compared with CAU (*P* = .85). This was neither moderated by an unmet sexual health need at baseline (*P* = .64) nor by the presence of a psychiatric disorder at baseline (*P* = .59).

**Conclusion:**

A substantial number of patients with HNC have unmet sexual health needs. SC targeting psychological distress does not reduce problems with sexuality in these patients. Interventions specifically targeting sexuality are recommended.

**Schutte LER, Melissant HC, Jansen F, et al. Effect of Stepped Care on Sexual Interest and Enjoyment in Distressed Patients with Head and Neck Cancer: A Randomized Controlled Trial. Sex Med 2021;9:100304.**

## Introduction

Sexual problems are highly prevalent in patients with cancer and include changes in sexual function, activity, and pleasure (eg, vaginal dryness, erectile and orgasm dysfunctions, decreased sexual desire, arousal, and enjoyment).^1^^,^^2^ These problems can lead to significant distress and are, besides other adverse (bio)psychosocial consequences (eg, pain, anxiety, fatigue), among the most negative influences of cancer and its treatment on quality of life.^1^, ^2^, ^3^ Even though the cancer is located outside the genital organs, patients with head and neck cancer (HNC) are at risk for developing intimacy issues or sexual problems.^4^^,^^5^ The disruption of physiological, psychological, and social functioning that accompanies HNC could all negatively impact sexuality directly, indirectly, and reciprocally.^1^^,^^2^ For example, treatment of patients with HNC often results in visible facial disfigurement (eg, scars or stoma in the neck), communication complications and other psychological and functional deficits (eg, problems with smell, speaking, and swallowing) that may interfere with intimate contact or sexual performance (eg, kissing or oral sex).^4^, ^5^, ^6^, ^7^, ^8^ Thus, sexuality of patients with HNC may be affected in a multidimensional manner.

However, sexuality is often overlooked, despite being an integral part of general health.^5^^,^^8^^,^^9^ Only a limited number of studies have investigated sexuality among patients with HNC, most of whom used a questionnaire module specifically developed for patients with HNC, with 2 questions that likely represent aspects of sexuality that are most immediately affected by cancer and cancer treatment: reduced interest in sex and reduced enjoyment of sex.^10^ These studies indicate that HNC and its treatment have a negative impact on these aspects of sexuality, especially immediately after oncological treatment, and particularly in those patients with high levels of distress, disrupted social functioning, extensive disfigurement, and advanced tumor stages.^7^^,^^8^^,^^11^^,^^12^ In addition, sexuality was listed by patients with HNC in the top 3 of the most bothersome domains of their life.^13^ Furthermore, over one-fifth of patients with HNC who underwent a total laryngectomy expressed that their need for supportive care targeting sexual problems was not satisfactorily met.^14^ These findings indicate that adequate screening and interventions are needed to help detect and address intimacy issues and sexual problems in patients with HNC.

A randomized controlled trial (RCT) was conducted to investigate efficacy of stepped care (SC) directed at psychological distress compared with care as usual (CAU) in Dutch HNC and lung cancer (LC) patients with psychological distress.^15^, ^16^, ^17^ This study also used the questionnaire module that includes the questions on sexual interest and enjoyment. The findings of this RCT showed that SC significantly reduced psychological distress and improved quality of life, particularly in patients with a psychiatric disorder.

Poor functioning in the general life domain (eg, low self-esteem, depression, and neuroticism) may negatively affect the marital and sexual domain in patients with cancer.^12^^,^^18^^,^^19^ Given the significant association between psychological distress and sexual problems,^8^^,^^12^^,^^18^^,^^19^ it is important to understand how these coexisting symptoms can be alleviated. Therapeutic interventions targeting sexuality can improve psychological wellbeing,^20^ but it is still unknown whether interventions targeting psychological distress also reduce sexual problems in patients with cancer.

Although SC in this study was not specifically directed at sexual problems, it is plausible that sexual interest and enjoyment may also improve, given that psychological distress decreased due to SC. The purpose of the present (post hoc) study was, therefore, to explore the effect of SC compared with CAU on the course of sexual interest and enjoyment, using data from the aforementioned RCT. Another purpose was to examine whether the effect of SC was moderated by having an unmet sexual health need and by the presence of a psychiatric disorder at baseline. It was hypothesized that SC targeting psychological distress also reduces problems with sexual interest and enjoyment among patients with HNC.

## Materials and methods

### Study Design and Population

In this study, analyses were performed using data of a parallel-group RCT on the efficacy of SC among HNC and LC patients with psychological distress.^16^ Patients with HNC and patients with LC who visited the outpatient clinic of the Amsterdam University Medical Centers (Amsterdam UMC), location VU University medical center (VUmc), between 2009 and 2013 for a follow-up consultation at least 1 month after curative treatment were randomly allocated (1:1) by an independent person to SC or CAU. Patients with HNC or LC were included in case they had psychological distress (based on validated cutoff points on the Hospital Anxiety and Depression Scale (HADS),^21^ ie, distress score >14 or a HADS anxiety or depression score >7), and completed treatment with curative intent at least 1 month previously. Patients were excluded in case of cognitive dysfunction, lack of motivation to undergo psychological treatment, current treatment for a depressive or anxiety disorder, treatment for a psychiatric disorder <2 months ago, high suicide risk, psychotic and manic signs, or insufficient knowledge of the Dutch language. Informed consent was obtained before any data collection. The study was approved by the Medical Ethics Committee of Amsterdam UMC, location VUmc, was registered in the Netherlands Trial Registration (NL1758)^15^ and conducted in accordance with the principles of the Declaration of Helsinki. More information on the eligibility criteria, randomization procedure, and sample size calculation can be found in previous publications.^15^^,^^16^

### Stepped Care Intervention

The SC program consisted of 4 steps. The first step was a watchful waiting period of 2 weeks during which it was agreed on not to start treatment, but to wait for further development, as symptoms may recover spontaneously. Step 2 was a guided self-help program via internet or a booklet which took 5 weeks. Patients were asked to describe what is important in their lives, to make a list of problems and concerns, and to categorize them into 3 categories, namely unimportant problems, important and amenable problems (these were solved using problem-solving techniques), and important but unsolvable problems. Trained coaches guided the patient through the process by means of short email or telephone contact. Step 3 contained 6 sessions of face-to-face problem-solving treatment by a nurse. The aim of problem-solving treatment is to identify problems that interfere with everyday functioning and that contribute to symptoms of depression and anxiety. The problem-solving treatment provides compensatory strategies aimed at bypassing the person's cognitive limitations and improving adaptive functioning. Finally, step 4 consisted of specialized psychological interventions (eg, cognitive behavioral therapy) by a psychologist or psychiatrist and/or psychotropic medication. All 4 steps focused on psychological distress and not specifically on sexuality. Patients who did not recover after a SC-treatment step (HADS anxiety or depression score remained above 7) proceeded to the next step in the SC program. A detailed description of the study design and SC program can be found elsewhere.^15^^,^^16^

### Data

All patient-reported outcome measures were collected at baseline (T0), after the SC-intervention period (time depended on duration of the SC program) or control period (4 months) (T1), and 3, 6, 9, and 12 months after T1, using paper and pencil or OncoQuest, a touch screen computer-assisted data collection system.^22^^,^^23^ As described previously^16^^,^^17^ 28% of patients recovered spontaneously after step 1, 35% did so after step 2, 9% after step 3, and 17% after step 4. On average, time between T0 and T1 was comparable.^16^

### Primary Outcome

The patient-reported outcome measure was the “sexuality” symptom subscale, part of the European Organization for Research and Treatment of Cancer, Quality of Life Questionnaire, HNC-specific module (EORTC QLQ-H&N35).^10^^,^^24^^,^^25^ This subscale contains 2 questions on sexual interest and enjoyment: “During the last week have you felt less interest in sex?” and, “During the last week have you felt less sexual enjoyment?”. Both items are scored on a four-point scale (“not at all”, “a little”, “quite a bit”, “very much”). The scores of these 2 items are averaged and then transformed into a scale ranging from 0 to 100, with higher scores implying less sexual interest and enjoyment. A score higher than 10 on this subscale indicates an unmet need for help in this domain (cutoff = 10).^25^ Cronbach's alpha in this study was 0.96.

### Other Outcomes

#### Sociodemographic and Clinical Variables

Information on age (continuous), gender (male, female), marital status (married/living together, unmarried/divorced/widow), years of education (continuous), and employment status (paid job, no paid job) was collected by means of self-report questionnaires. Information about tumor location (lip/oral cavity/oropharynx, hypopharynx/larynx, other), tumor stage (I, II, III, IV), and type of treatment (surgery, radiotherapy, chemoradiation, surgery + radiotherapy, surgery + chemoradiation, surgery + chemotherapy) was obtained from medical records.

#### Psychological Distress

The HADS is a 14-item psychometrically sound, self-assessment scale for measuring distress (total HADS score) with 2 subscales, anxiety (HADS-A) and depression (HADS-D), developed for non-psychiatric patients. The total HADS score ranges from 0 to 42, the subscales from 0 to 21, where higher scores represent more distress.^21^^,^^26^ HADS at baseline was assessed by telephone or by means of OncoQuest.^22^^,^^23^ Cronbach's alpha of the HADS in this study was 0.86 (HADS-A), 0.86 (HADS-D), and 0.90 (HADS-total). The presence of a psychiatric (depressive or anxiety) disorder was assessed by telephone using the Composite International Diagnostic Interview (CIDI),^27^ a comprehensive, structured interview designed for the assessment of mental disorders such as anxiety and depression by trained lay interviewers.

#### Health-Related Quality of Life

A global quality of life (QOL) scale and 5 functional scales (physical, role, emotional, cognitive, and social) were assessed with the EORTC Quality of Life Questionnaire Core 30 questions (EORTC QLQ-C30); a cancer-specific questionnaire. All scales and single items were linearly transformed into a score from 0 to 100, with a higher score indicating a higher level of functioning. The questionnaire has shown adequate psychometric properties in cancer patient populations.^10^^,^^28^ Cronbach's alpha in this study ranged from 0.70 (nausea and vomiting) to 0.89 (emotional functioning).

### Statistical Analyses

All analyses were performed using SPSS version 20 (IBM Corp., Armonk, NY). Sociodemographics, clinical characteristics, HADS scores, CIDI diagnosis, and QOL measurements of the study sample (at baseline) were summarized using descriptive statistics. Independent samples t-tests and χ^2^ tests were used to examine whether randomization of the patients with HNC had resulted in a balanced distribution of sociodemographic and clinical characteristics, global QOL and all functioning domains across SC and CAU. Independent samples t-tests were also used to measure differences between SC and CAU in sexual interest and enjoyment at each time point. An absolute difference in sexual interest and enjoyment ≥10% of the instrument range was considered clinically meaningful.^29^ A linear mixed model (LMM) was used to compare differences in the course of sexual interest and enjoyment between SC and CAU, with fixed effects for intervention, time point and their two-way interaction, and a random intercept for subject. To control for a potential confounding effect of differences in sexual interest and enjoyment at baseline between the 2 interventions, an adjusted LMM was used where sexual interest and enjoyment at baseline was added as a fixed covariate to the previous model. In addition, 2 other adjusted LMMs were used to investigate the effect of SC on the course of sexual interest and enjoyment with 2 potential moderators: a psychiatric disorder at baseline (based on the CIDI) and having an unmet sexual health need at baseline (sexuality score > 10),^25^ using a random intercept for subjects, fixed effects for intervention, time point, moderator, and all two-way and three-way interactions. For all analyses missing data were excluded analysis by analysis rather than listwise and a *P* value of < .05 was considered statistically significant. The data were analyzed on an intention-to-treat basis.

## Results

### Sample Characteristics

**Figure 1:**
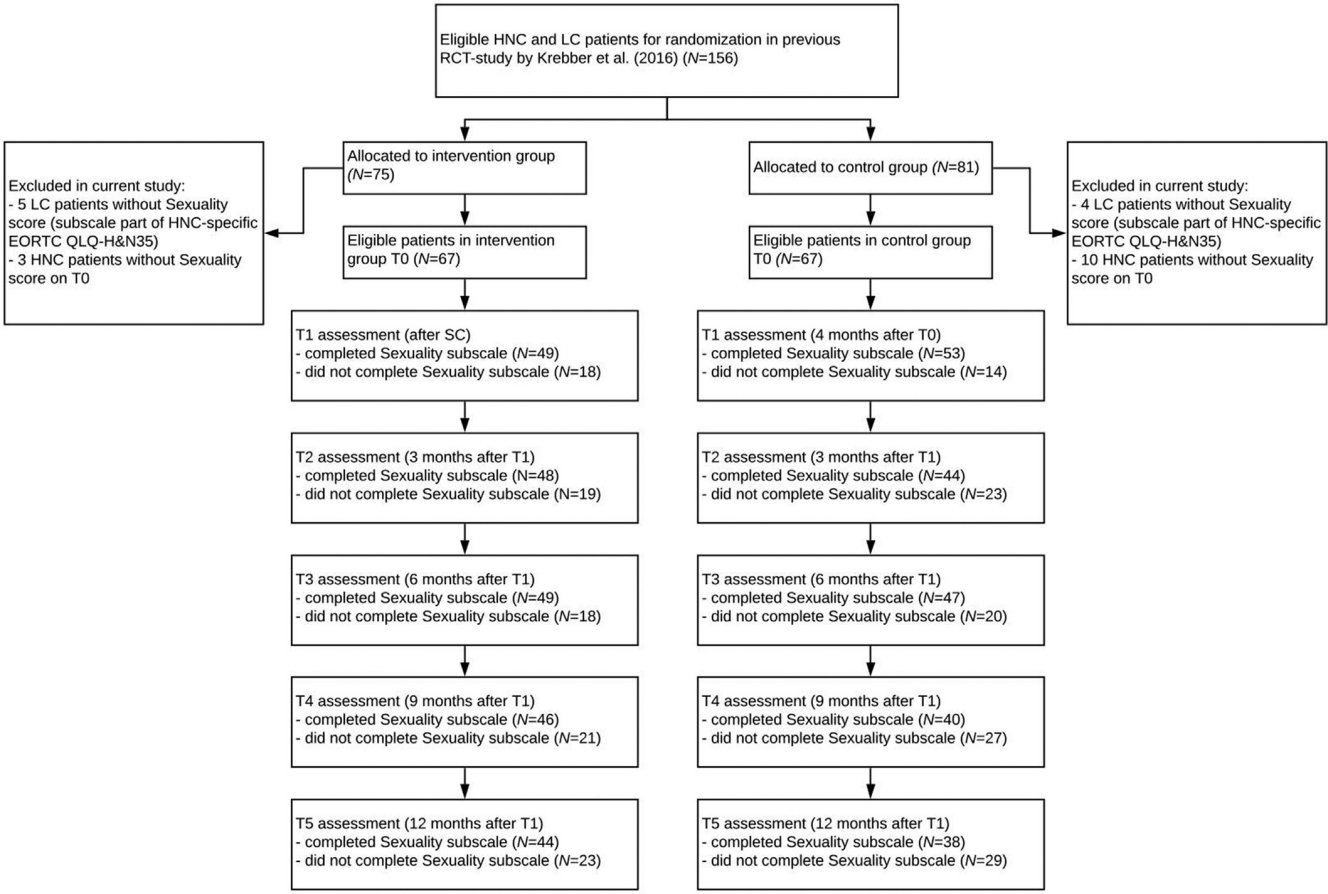
CONSORT flow diagram.

Sexuality data were unavailable for all 9 patients with LC (Figure 1). Of the remaining 147 patients with HNC, 134 patients (67 in the SC and 67 in the CAU group), of whom 64.2% male and 35.8% female, provided a baseline score (T0) on the sexuality subscale. Patients in the SC group scored significantly better on sexual interest and enjoyment at baseline (T0): 39.5 versus 51.7; *P* = .040. They also scored significantly better on the HADS-total (17.5 versus 19.5; *P* = .030), HADS-D (8.28 versus 9.96; *P* = .009), and the QLQ-C30 social functioning subscale (71.9 versus 58.7, *P* = .004), see Table 1.

**Table 1:** Patient characteristics at baseline

Patient characteristic	Intervention (*N* = 67)	Control (*N* = 67)	Total (*N* = 134)	*P*-value
Age (mean, SD)	62.5 (8.5)	61.1 (9.9)	61.8 (9.2)	.37
Gender				.86
Men	44 (65.7%)	42 (62.7%)	86 (64.2%)	
Women	23 (34.3%)	25 (37.3%)	48 (35.8%)	
Paid job				.86
Yes	22 (32.8%)	24 (35.8%)	46 (34.3%)	
No	45 (67.2%)	43 (64.2%)	88 (65.7%)	
Marital status				.57
Married/living together	49 (73.1%)	45 (67.2%)	94 (70.1%)	
Unmarried/divorced/widowed	18 (26.9%)	22 (32.8%)	40 (29.9%)	
Years of education				.29
5-10	33 (49.3%)	24 (35.8%)	57 (42.5%)	
11-16	30 (44.7%)	38 (56.7%)	68 (50.8%)	
17-21	4 (6.0%)	5 (7.5%)	9 (6.7%)	
Tumor location				.079
Lip/oral/cavity/oropharynx	29 (43.3%)	42 (62.7%)	71 (53%)	
Hypopharynx/larynx	21 (31.3%)	14 (20.9%)	35 (26.1%)	
Other head and neck cancers	17 (25.4%)	11 (16.4%)	28 (20.9%)	
Tumor stage				.22
Unknown	7 (10.4%)	2 (3.0%)	9 (6.7%)	
I	13 (19.4%)	17 (25.4%)	30 (22.4%)	
II	15 (22.4%)	9 (13.4%)	24 (17.9%)	
III	9 (13.4%)	13 (19.4%)	22 (16.4%)	
IV	23 (43.3%)	26 (38.8%)	49 (36.6%)	
Tumor treatment				**.005**
Surgery	11 (16.4%)	19 (28.4%)	30 (22.4%)	
Radiotherapy	22 (32.8%)	12 (17.9%)	34 (25.4%)	
Chemoradiation	5 (7.5%)	18 (26.9%)	23 (17.2%)	
Surgery + radiotherapy	25 (37.3%)	14 (20.9%)	39 (29.1%)	
Surgery + chemoradiation	4 (6.0%)	3 (4.4%)	7 (5.2%)	
Surgery + chemotherapy	0	1 (1.5%)	1 (0.7%)	
Time since treatment				.65
>7 months	26 (38.8%)	23 (34.3%)	49 (36.6%)	
7-12 months	10 (14.9%)	14 (20.9%)	24 (17.9%)	
>12 months	31 (46.3%)	30 (44.8%)	61 (45.5%)	
Anxiety or depression disorder (CIDI)				.42
Yes	14 (20.9%)	19 (28.4%)	33 (24.6%)	
No	53 (79.1%)	48 (71.6%)	101 (75.4%)	
HADS (mean, SD)				
Total	17.5 (5.2)	19.5 (5.8)	18.5 (5.6)	**.030**
Depression	8.28 (3.6)	9.96 (3.7)	9.62 (3.6)	**.009**
Anxiety	9.18 (3.6)	9.58 (3.7)	9.38 (3.7)	.53
EORTC QLQ-C30 (mean, SD)				
Global quality of life	59.5 (19.8)	55.5 (19.5)	57.46 (19.7)	.24
Physical functioning	71.6 (20.9)	70.7 (20.8)	71.16 (20.8)	.79
Role functioning	62.2 (26.7)	55.5 (26.0)	58.83 (26.5)	.14
Emotional functioning	58.3 (26.1)	56.3 (22.6)	57.30 (24.3)	.65
Cognitive functioning	71.4 (27.3)	70.6 (24.5)	71.02 (25.9)	.87
Social functioning	71.9 (25.2)	58.7 (27.1)	65.30 (26.9)	**.004**
EORTC QLQ-H&N35 (mean, SD)				
Sexuality subscale	39.6 (34.6)	51.7 (33.6)	45.65 (34.5)	**.040**
Unmet sexual health need (sexuality subscale > 10)				.068
Yes	46 (68.7%)	56 (83.6%)	102 (76.1%)	
No	21 (31.3%)	11 (16.4%)	32 (23.9%)	

Significant differences (*P* < .05) are presented in bold font.

SD = standard deviation; CIDI = Composite International Diagnostic Interview; HADS = Hospital Anxiety and Depression Scale; EORTC QLQ-C30 = European Organization for Research and Treatment of Cancer, Qualify of Life Questionnaire; H&N35 = head and neck specific module.

### Effect of SC on Sexual Interest and Enjoyment After Intervention

When comparing differences in sexual interest and enjoyment between SC and CAU per time point, patients in the SC group scored statistically better after intervention (T1) (34.7 versus 55.7; *P* = .004). However, when correcting for the baseline difference in sexual interest and enjoyment, no significant within-subjects change from baseline (T0) to postintervention (T1) was found (*P* = .37).

### Effect of SC on the Course of Sexual Interest and Enjoyment

**Figure 2:**
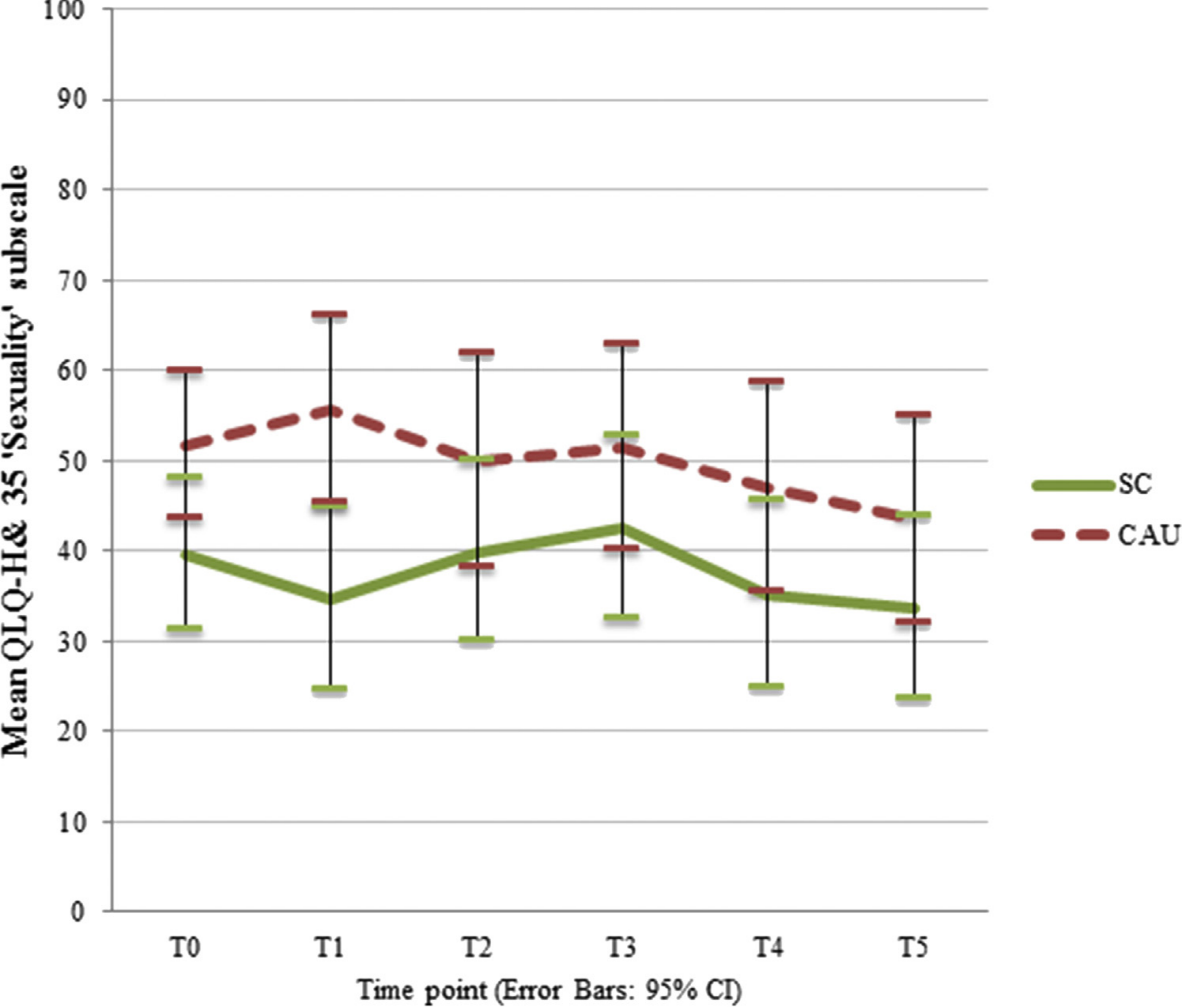
Effect of SC (stepped care) and CAU (care as usual) on sexual interest and enjoyment from T0 (preintervention) to T5 (12 months follow-up), corrected for between-group baseline differences, with 95% confidence intervals. Higher scores represent less sexual interest and enjoyment.

LMM corrected for the between-group baseline difference in sexual interest and enjoyment showed that the course of sexual interest and enjoyment over time points did not differ between SC and CAU groups (time point ∗ intervention: *P* = .85), see Figure 2. Sexual interest and enjoyment scores ranged between 33.7 (T5) and 42.5 (T3) in de SC group, and between 43.4 (T5) and 55.7 (T1) in the CAU group.

### Factors Moderating the Effect of SC on the Course of Sexual Interest and Enjoyment

Of all patients, 76.1% had an unmet sexual need at baseline, and 24.6% had a psychiatric disorder, see Table 1. Neither having a psychiatric disorder at baseline (time point ∗ intervention ∗ psychiatric disorder: *P* = .59) nor an unmet sexual health need at baseline (time point ∗ intervention ∗ sexuality: *P* = .64) moderated the effect of SC on the course of sexual interest and enjoyment postintervention^a^.

## Discussion

A substantial number of patients with HNC were found to have an unmet sexual health need. SC did not reduce problems with sexual interest and enjoyment at any of the follow-up measurements compared with CAU, after correcting for baseline differences. In addition, moderator analyses showed that patients with an unmet sexual health need at baseline and patients with a psychiatric disorder at baseline had no greater benefit from SC. These findings suggest that mere alleviation of illness-related psychological distress through SC is insufficient to effectively improve sexual interest and enjoyment in patients with HNC, implying that interventions specifically targeting sexuality are needed for (HNC) patients who experience sexual problems.

The latter suggestion is supported by a study of Hummel et al^30^ that demonstrated that Internet-based cognitive behavioral therapy directed at sexual functioning in breast cancer survivors with sexual dysfunction significantly reduced sexual problems and body image concerns. Online therapy was guided by a psychologist and specifically tailored to the sexual problems of each patient. Another study evaluated a telephone counseling intervention to improve psychosocial outcomes including sexual dysfunction in patients with early-stage breast cancer. Sexual functioning only improved in the intervention group, where sexual functioning was deliberately targeted.^31^ The active control group (without sexual counseling) showed no improvement in sexual functioning.

Considering these findings, it can be concluded that sexual health care needs of patients with HNC should not be underestimated and that interventions solely targeting psychological distress do not coalleviate sexual problems in patients with cancer.

Interventions directed at sexuality address both psychological and sexual problems and possibly also their interaction. Thus, an integral approach for mental health care in patients with cancer is recommended where psychological and sexual problems are targeted simultaneously.

Strengths of this study are the randomized controlled design, the long follow-up period, inclusion of an active control group, and use of LMM which enables use of all collected data. A major limitation was that sexuality was assessed with 2 items only because the RCT from which the data were adopted did not specifically focus on sexuality.^16^ Validity of these items and sensitivity to change may be limited.

Future studies may incorporate questions on sexual activity, and a more comprehensive and valid measure of sexual function (assessing problems as well as wellbeing) in interventions for patients with (HNC) cancer and their partners. Such a sexual health questionnaire is currently being developed in accordance with EORTC guidelines.^32^^,^^33^ When the psychometric qualities of this measure are established, it can be used to evaluate interventions or to tailor and monitor care.

Given the substantial unmet sexual health need and the importance of sexuality to general health, patients with HNC and their partners should be asked whether they experience sexual problems and want referral for help.^34^, ^35^, ^36^ In this regard, a stepped care format might be useful to target sexual problems because of its (cost) effectiveness and accessibility. Patients would be routinely screened for sexual symptoms and, when appropriate, start with low cost (self-)management (eg, eHealth) before stepping up to more intensive treatment (eg, sexual counseling) if symptoms are not alleviated. Future research is warranted that investigates the effectiveness of such a stepped care format directed at both psychological and sexual problems in patients with HNC.

## Conclusion

A substantial number of patients with HNC have unmet sexual health needs. SC targeting psychological distress does not reduce problems with sexual interest and enjoyment in these patients. Interventions specifically targeting sexuality are needed for patients who experience sexual problems.

## Statement of authorship

All authors contributed to conception and design. Analysis of data was performed by Laura E.R. Schutte, Heleen C. Melissant, and Femke Jansen.Interpretation of data was carried out by Laura E.R. Schutte, Heleen C. Melissant, Femke Jansen, and Ellen Laan. Drafting the article was contributed by Laura E.R. Schutte and Ellen Laan. Revising it critically for important intellectual content was carried out by all authors. Final approval of the manuscript was done by all authors.

## Footnotes

a Neither gender nor tumor characteristics (location and stage) affected the main findings.
